# Commentary: Structural and functional features of central nervous system lymphatic vessels

**DOI:** 10.3389/fnins.2015.00485

**Published:** 2015-12-22

**Authors:** James J. Bradstreet, Marco Ruggiero, Stefania Pacini

**Affiliations:** ^1^The Brain Treatment CenterBuford, GA, USA; ^2^Faculty for Autism Collaboration & Education, Western University of Health SciencesPomona, CA, USA; ^3^Dream Master LaboratoryGilbert, AZ, USA; ^4^Department of Experimental and Clinical Medicine, University of FlorenceFirenze, Italy

**Keywords:** autism, meninges, lymphatics, ultrasonography, neurodevelopment

Autism spectrum disorders (ASD) represent an apparent pandemic threat to child development with the current CDC data documenting ASD affecting over 2% of U.S. males of school age ([CDC] Developmental Disabilities Monitoring Network Surveillance Year 2010 Principal, 2014). ASD are likely a heterogeneous group of disorders with genetic and environmental causes resulting in similar phenotypes. Genetic contributions to autism are extremely heterogeneous and may involve synaptic formation and maturation. Thus, multiple genes involved in the formation, specification, and maintenance of synapses have been identified as risk factors for ASD development (Hahn et al., 2013). Also the rate of brain growth in the first 2 years of life may contribute to ASD. Although abnormally enlarged brain volumes and increased rates of brain growth during early childhood are observed only in a minority of ASD children, nevertheless there is evidence of abnormalities in posterior lobes and posterior brain networks during the first 2 years of life in ASD (Lainhart, 2015).

If the etiology of ASD is still mysterious, there is a growing consensus for the role of neuroinflammation in ASD pathogenesis (Fatemi et al., 2012), and the recent publication in Nature of the existence of the previously unknown meningeal lymphatic system, invites a closer evaluation of its potential significance to brain development (Louveau et al., 2015).

In 1983, Aarli speculated the fluid of the brain traveled along mysterious perivascular channels, and noted that immunological challenges could affect the integrity of the blood brain barrier (Aarli, 1983). It was then accepted that the exit pathway for immune cells trafficking through the CNS was *via* the arachnoid granulations. The new study in Nature provides us with a secondary pathway leading to deep cervical lymph nodes.

We concur with the comment (Louveau et al., 2015) that; “the presence of a functional and classical lymphatic system in the central nervous system suggests that current dogmas regarding brain tolerance and the immune privilege of the brain should be revisited.”

This feature is critically important with regard to the observations of immunological dysregulation in autism, which intersect with observations of increased extra-axial CSF (EAF) in the autism population. MRI scans were used (Shen et al., 2013) to evaluate the EAF of infants born into families with an existing autistic child. They were able to predict the future onset and severity of autism based on the findings of early and persistent increases in EAF. In a similar way, we were able to demonstrate increased EAF using transcranial ultrasonography and we too observed a correlation between increased severity of autistic symptoms and increased EAF scores (Bradstreet et al., 2014). The mechanisms responsible for such an increased EAF, however, remained unknown. The newly discovered meningeal lymphatic system might help explaining how immunological dysfunctions and peripheral chronic infection/inflammation may affect the meninges and, consequently, brain development.

Several evidences point to a connection between meninges and abnormal CNS development. Meningeal cells are involved in cortical development (Dragunow, 2013), and meningeal alterations in mice modeling ASD-like behaviors contribute to incorrect neurogenesis during development (Mercier et al., 2011). In 2012, Zarbalis et al. demonstrated that meningeal defects alter migration of cortical interneurons in a mouse model, thus further stressing the role of the meninges in the establishment of proper neuronal interconnections (Zarbalis et al., 2012).

The observations of increased EAF in the autism population with these new observations of a central lymphatic system connected to cervical lymph nodes, should direct more attention to the role of meningeal lymphatics in the pathogenesis of ASD. It further begs the question: “could inflammation-associated deficits in meningeal lymph drainage be the culprit for the observed increased EAF?”

Intersecting the increased EAF volume observations in ASD with the EAF drainage to deep cervical lymph nodes draws our attention to the pathogenetic potential of chronic infections leading to inflammation and subsequent deficit in lymphatic drainage.

Supporting the role of chronic infection/inflammation in ASD pathogenesis, multiple polyomaviral infections were observed to be significantly more common in the post-mortem brains of ASD individuals (Lintas et al., 2010) and ASD individuals show immune transcriptome alterations in the temporal cortex that seem to indicate immune dysregulation with consequent inflammation (Garbett et al., 2008). Piras et al. (2014) correlated anti-brain antibodies with specific deficits in ASD, thus reinforcing the notion that chronic inflammation is a common denominator that may lead to EAF increase because of impaired meningeal lymphatic drainage.

The existence of a classical lymphatic system in the CNS might also explain the nature of the lesions in the brains of autistic subjects, which we observed with ultrasonography and designated “cortical dysplasia” (Bradstreet et al., 2014). Inefficient drainage with focal accumulation of CSF in certain areas of the cortex might explain the hypoechogenic appearance of focal “patches,” which we consistently observed in autistic subjects. Accumulation of fluid that typically appears hypoechogenic in ultrasonography, might thus disrupt neuronal/glial networking by increasing the distance between cells and by increasing extracellular pressure on cells with consequent alteration of gene expression trough modification of the cytoskeleton (Knöll, 2010). Since Stoner et al. (2014) also observed “patches” of decreased transcription in autism related brain bank specimens, it is tempting to speculate that such alterations of gene expression may be associated with the accumulation of EAF and its effects on neuronal and glial function.

Finally, transcranial ultrasonography deserves more attention as a harmless and low-cost means of evaluating CSF fluid volumes and stratifying ASD children potentially at-risk for chronic CNS inflammatory disorders. Transcranial ultrasonography enables reproducible evaluation of EAF by measuring the distances between the arachnoid membrane and the cortical pia layer (subarachnoid space), and may thus help establishing the degree of meningeal lymphatic drainage deficit. Since the measures can be easily repeated, the technique could be used for monitoring the progression of the disease or for objectively assessing the efficacy of treatments.

In conclusion, the observation by Louveau et al. (2015) leads us to hypothesize that meningeal lymphatic drainage deficit due to peripheral chronic infection/inflammation may be responsible for increased EAF and cortical dysplasia in ASD individuals and, possibly, for some of the symptoms typical of the disorder.

## Conflict of interest statement

The authors declare that the research was conducted in the absence of any commercial or financial relationships that could be construed as a potential conflict of interest.
